# Effects of Testosterone and Its Major Metabolites upon Different Stages of Neuron Survival in the Dentate Gyrus of Male Rats

**DOI:** 10.3390/biom15040542

**Published:** 2025-04-07

**Authors:** Mark D. Spritzer, Ethan A. Roy, Kelsey M. K. Calhoun, Zachary E. Schneider-Lynch, Leslie Panella, Charlotte Michaelcheck, April Qian, Evan D. Kelly, Hadley Barr, Emma Hall, Blaine Cunningham, Hieu H. M. Nguyen, Dani Xu, Jennifer M. Barker, Liisa A. M. Galea

**Affiliations:** 1 Department of Biology, McCardell Bicentennial Hall, Middlebury College, Middlebury, VT 05753, USA; ecunningham@middlebury.edu; 2 Program in Neuroscience, McCardell Bicentennial Hall, Middlebury College, Middlebury, VT 05753, USA; ethanroy@stanford.edu (E.A.R.); kelsey@vtresiliency.com (K.M.K.C.); zachschneiderlynch@gse.harvard.edu (Z.E.S.-L.); lesliepanella@gmail.com (L.P.); michaelcheck.charlotte@mayo.edu (C.M.); hadley_barr@dfci.harvard.edu (H.B.); emma.hall@vailhealth.org (E.H.); hmhn2@cam.ac.uk (H.H.M.N.); 3 Program in Molecular Biology and Biochemistry, McCardell Bicentennial Hall, Middlebury College, Middlebury, VT 05753, USA; qian.ap@northeastern.edu (A.Q.); edkelly@middlbury.edu (E.D.K.); dxu@middlebury.edu (D.X.); 4 Department of Biology, Faculty of Science and Technology, Douglas College, Coquitlam, BC V3L 5B2, Canada; barkerj1@douglascollege.ca; 5 Treliving Family Chair in Women’s Mental Health, Centre for Addiction and Mental Health, Toronto, ON M5T 1R8, Canada; liisa.galea@camh.ca; 6 Department of Psychiatry, University of Toronto, Toronto, ON M5T 1R8, Canada

**Keywords:** adult neurogenesis, cell proliferation, dentate gyrus, hippocampus, testosterone, estradiol, dihydrotestosterone, androgen, estrogen, rat

## Abstract

Testosterone has been shown to enhance hippocampal neurogenesis through increased cell survival, but which stages of new neuron development are influenced by testosterone remains unclear. Therefore, we tested the effects of sex steroids administered during three different periods after cell division in the dentate gyrus of adult male rats to determine when they influence the survival of new neurons. Adult male rats were bilaterally castrated. After 7 days of recovery, a single injection of bromodeoxyuridine (BrdU) was given on the first day of the experiment (Day 0) to label actively dividing cells. All subjects received five consecutive days of hormone injections during one of three stages of new neuron development (days 1–5, 6–10, or 11–15) after BrdU labeling. Subjects were injected during these time periods with either testosterone propionate (0.250 or 0.500 mg/rat), dihydrotestosterone (0.250 or 0.500 mg/rat), or estradiol benzoate (1.0 or 10 µg/rat). All subjects were euthanized sixteen days later to assess the effects of these hormones on the number of BrdU-labeled cells. The high dose of testosterone caused a significant increase in the number of BrdU-labeled cells in the hippocampus compared to all other groups, with the strongest effect caused by later injections (11-15 days old). In contrast, neither DHT nor estradiol injections had any significant effects on number of BrdU-labeled cells. Fluorescent double-labeling and confocal microscopy reveal that the majority of BrdU-labeled cells were neurons. Our results add to past evidence that testosterone increases neurogenesis, but whether this involves an androgenic or estrogenic pathway remains unclear.

## 1. Introduction

In human males, total testosterone begins to decline by age 30 at a rate of about 2% per year [1,2,3], and incidence of hypogonadism (serum testosterone levels consistently below 325 ng/dL) is about 20% in males 60–69 years old and about 50% in males over 80 [2]. Given that this decline in testosterone parallels the age-related decline in cognitive ability, there has been much speculation that there may be a causal link between the two [4]. Despite some inconsistencies [5,6], past research indicates that testosterone levels in human males correlate with improved ability on a variety of hippocampus-dependent cognitive tasks [7,8,9,10]. Androgen deprivation therapy given as a treatment for prostate cancer impairs spatial memory [11,12,13,14,15] and testosterone treatment given to hypogonadal young to middle-aged males both resulted in improvements in cognitive ability [16]. Experiments with rodent models confirm that testosterone can enhance hippocampus-dependent memory [17]. Specifically, current evidence indicates that testosterone influences spatial working memory and some forms of long-term memory. In a variety of maze tasks, castration impaired spatial working memory [18,19,20,21,22,23], and testosterone replacement given to castrated male rats improved spatial working memory [21,24]. Additionally, experiments with the object location task have consistently shown that testosterone improves long-term spatial memory [25,26,27], and one study showed that testosterone improved 24 h retention on a passive avoidance task [28].

The mechanism by which testosterone influences hippocampus-dependent memory remains unknown, but one possible mechanism involves changes in adult neurogenesis. Adult neurogenesis refers to the proliferation, migration, differentiation, and integration of new neurons within the adult brain [29]. Neurogenesis within the adult brain occurs within the subgranular zone (SGZ) of the dentate gyrus region of the hippocampus and the subventricular zone (SVZ) of the lateral ventricles [30]. Some researchers have documented adult neurogenesis in other regions of the brain, but current empirical evidence indicates the dentate gyrus and SVZ contain the majority of adult neuronal stem cells in mammals [31]. Neurogenesis occurs in the SGZ throughout adult life among most mammalian species studied to date [32], and numerous post-mortem studies have shown that neurogenesis also occurs in the human hippocampus throughout adult life [33,34,35,36], though there is variation with age [37,38].

Cells that divide within the SGZ migrate a short distance into the granule cell layer (GCL) of the dentate gyrus, where they mature into either neurons or glial cells [39,40,41]. Young neurons extend their axons from the GCL to form connections with CA3 pyramidal cells in the hippocampus [42,43]. Young hippocampal neurons exhibit increased Ca^2+^ conductance, enhanced excitability, and a lower threshold for the induction of long-term potentiation (LTP) than do mature neurons [44,45,46], which may make them a particularly good substrate for memory formation [47]. Experiments with rodents have shown that blocking hippocampal neurogenesis impairs various forms of spatial memory [48,49,50,51,52,53,54,55], whereas experimentally enhancing adult neurogenesis improves spatial memory [56]. Thus, there is evidence that adult neurogenesis has functional importance in the adult brain. Interestingly, one study found that blocking neurogenesis impaired spatial memory in male rats but not female rats, suggesting a modulatory role for sex steroids [55].

The effects of endogenous and environmental factors on adult neurogenesis depend upon the age of the newly proliferated cells. Cells in the brain can be birth-dated by injecting animals with a thymidine analog like 5-bromo-2′-deoxyuridine (BrdU) or using endogenous markers of specific stages of the cell cycle [57]. BrdU can be used to measure both the number of new cells produced (i.e., cell proliferation) and the number of cells surviving to specific time points (i.e., cell survival). The cell cycle takes approximately 24 h in the GCL [58]. Proliferating cells incorporate BrdU during the S phase of the cell cycle and then may undergo multiple cell divisions. BrdU can be detected in actively proliferating cells in the GCL up to four days after BrdU injection [59]. This means that manipulations up to 4 days after BrdU injection can influence cell proliferation, whereas after this period any manipulations will influence cell survival.

Using BrdU injections, it was shown that hippocampal-dependent learning on the Morris water maze enhanced neurogenesis among cells that were 6–10 days old at the time of training, but not among cells that were 1–5 or 11–15 days old in rats [60,61]. This result is supported by a number of other studies with rats showing that training on hippocampus-dependent tasks specifically enhances neurogenesis among relatively young cells (3–11 days old) but not among older cells (10–14 days old) [62,63,64,65,66]. One recent study found that cells that were 7 days old at the time of learning in the Morris water maze were required for memory reconsolidation four weeks later when rats were re-exposed to the water maze [67]. These results suggest that there may be a critical period when hippocampal neurogenesis contributes to learning and memory. For male rats, this period seems to be when cells are approximately 4–10 days old, which corresponds with the final stages of cell migration and early axon extension [42,68,69].

Sex steroids, in general, influence adult neurogenesis both in the SVZ and the dentate gyrus [70,71,72]. There is also considerable evidence that testosterone, specifically, influences neurogenesis within the dentate gyrus [73]. Among adult male rats and mice, castration had no effect on cell proliferation within the dentate gyrus [74,75,76] but caused a significant decrease in the survival of new neurons that were 15–30 days old [74,77,78]. Testosterone replacement or supplementation have no effect on cell proliferation in the dentate gyrus of adult male rodents [79,80,81,82,83,84], whereas prolonged exposure to testosterone (30–35 days) causes a significant increase in neurogenesis in the dentate gyrus compared to castrated control rats [74,82,85]. Surprisingly, shorter periods (15–21 days) of testosterone replacement did not increase neurogenesis relative to castrated control rats [78,79,80]. Testosterone dose is another key variable, as 30 days of injections at high doses (0.500–1.00 mg/rat) increased neurogenesis, whereas a lower dose (0.250 mg/rat) did not [74]. One goal of the current experiment was to assess the effects of hormone dose upon the early stages of neuron maturation in the dentate gyrus.

Many of the effects of testosterone occur via its two main metabolites, estradiol and dihydrotestosterone (DHT), and there is considerable evidence that testosterone enhances neurogenesis though an androgen-dependent pathway. Prolonged DHT injections (30–37 days) given to male rats or mice increased hippocampal neurogenesis, whereas prolonged estradiol injections had no significant effect on neurogenesis [74,85,86,87]. Fifteen days of estradiol injections also had no effect on hippocampal neurogenesis among male rats [88]. However, estradiol injections increased neurogenesis among adult male meadow voles during the axon extension phase of neural development (6–10 after cell birth) and not during earlier (1–5 days) or later (11–15 days) periods of development [89]. This finding suggests that the time of exposure to sex steroid hormones during new neuron maturation is critical for the promotion of neurogenesis.

Past experiments suggest that testosterone does not influence cell proliferation but enhances cell survival in the GCL of the dentate gyrus [74,78]. The goals of the current experiment were to clarify whether or not testosterone can influence the survival of cells during the early stages of neural development, and if so, whether these effects occur via an androgen- or estrogen-dependent pathway. We followed the same protocol as Ormerod et al. (2004) to assess the effects of sex steroids upon the early stages of adult neurogenesis among castrated adult male rats [89]. Specifically, we tested the effects of testosterone, estradiol, and DHT on cells that were in three different stages of new neuron development: (1) cell proliferation, differentiation, and early migration (days 1–5); (2) neurite growth and late cell migration (days 6–10); and (3) neuron maturation (days 11–15) [41,90]. Based on past findings [74,78], we expected that testosterone would exert its survival-promoting effects primarily in the latter two stages of new neuron development.

## 2. Materials and Methods

### 2.1. Subjects

Adult male Sprague–Dawley rats (approximately 60 days old) were obtained from Charles River Laboratories (St. Corustant, QC, Canada). Animals were individually housed in opaque polystyrene bins (21 cm × 42 cm × 21 cm) with Tek-Fresh bedding (Harlan Laboratories, Indianapolis, IN, USA). Animals were housed on a 12 h light–dark cycle (lights on at 0700 h). Animals had access to fresh water in glass water bottles and soy-free rodent chow (Harlan Teklad diet 2020X, Inotiv, Inc., Lafayette, IN, USA) throughout the experiments. All procedures used in this experiment were approved by the Middlebury College Animal Care and Use Committee and in accordance with the ethical guidelines established by the National Institutes of Health.

After their arrival at the Middlebury College vivarium, rats were given one week to acclimate before being bilaterally castrated. Surgeries were conducted using aseptic procedures under isoflurane anesthesia in oxygen at 3.5–4.0% during induction and 2.0–2.5% during maintenance. Before surgery began, rats were weighed and given an injection of ketoprofen (5 mg/kg body mass s.c.) to act as an analgesic. Both testes were removed through a small incision at the posterior end of the scrotum and double ligated using chromic gut suture material (Ethicon, Somerville, NJ, USA). The muscular layer was sutured closed using chromic gut sutures and the skin layer was closed with ethilon sutures (Ethicon, Cincinnati, OH, USA). Immediately after surgery, a topical antibiotic (Terramycin, Zoetis, Kalamazoo, MI, USA) was applied to the site of incision, and the rats were given a subcutaneous injection of lactated Ringer’s solution (10 mL/kg) as fluid replacement. Twenty-four hours after the surgeries, rats were given a second injection of ketoprofen (5 mg/kg). For one week following surgery, the rats were weighed daily and the incisions checked to ensure normal recovery. Rats were handled for approximately 4 min during the last four days of recovery to acclimate to the experimenters.

### 2.2. Procedure

**Figure 1:**
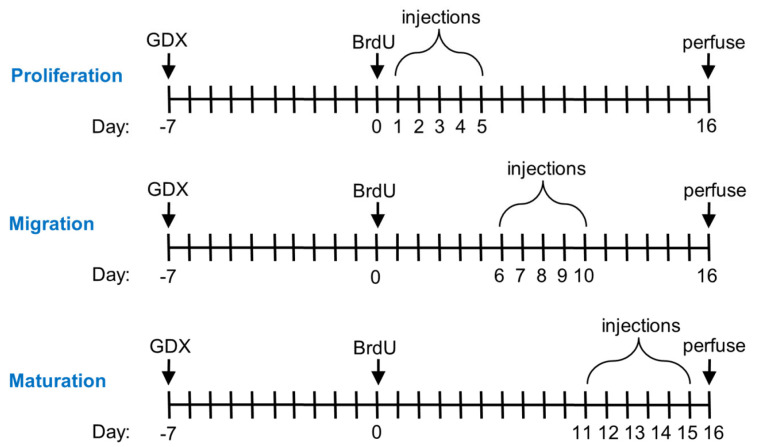
Timeline for the experiments. Castration surgeries (GDX) occurred 7 days prior to BrdU injections (200 mg/kg). Oil (control) or hormone (T, DHT, E2) injections occurred during different 5-day time periods of neuronal development. On day 16, rats were euthanized and perfused with paraformaldehyde and saline before brain tissue was collected for histological analyses.

This study consisted of three experiments designed to assess the effects of testosterone propionate (T), dihydrotestosterone (DHT), and estradiol benzoate (E2) on the stages of adult neurogenesis. For each experiment, the castrated male rats were divided into nine groups (*n* = 5–8/group) that received subcutaneous injections of either 0.1 mL sesame oil (control groups) or a steroid hormone dissolved in 0.1 mL of sesame oil on days 1–5, 6–10, or 11–15 of neural development (Figure 1). Injections began one week after surgeries, a time point when testosterone is no longer detectable in circulation [74]. Doses of T and DHT were 0.250 and 0.500 mg/rat. These doses were previously shown to enhance hippocampal neurogenesis in male rats, and the doses resulted in high physiological levels of circulating testosterone [74]. Doses of E2 were 1.0 μg/rat and 10 μg/rat. The high dose resulted in circulating estradiol levels that are 10-fold greater than physiological levels for a male rat [74], so the lower dose was assumed to be close to physiological levels. Twenty-four hours prior to starting injections, subjects were given an intraperitoneal injection of 200 mg/kg of the thymidine analog 5-Bromo-2′deoxyuridine (BrdU), which served to label dividing cells. BrdU was prepared by dissolving 20 mg/mL in a 0.9% saline solution buffered with 0.7% NaOH and filtered through a 0.22 µm syringe filter into sterile injection vials.

Sixteen days after BrdU injections, all subjects were euthanized with a lethal dose (120 mg/kg i.p.) of sodium pentobarbital (Fatal+; Vortech Pharmaceuticals, Dearborn, Michigan). Rats were transcardially perfused using 60 mL of 0.9% saline followed by 120 mL of 4% paraformaldehyde (pH = 7.4) using a syringe pump. The brains were then extracted and post-fixed in 4% paraformaldehyde overnight. They were then saturated with 30% sucrose in 0.1 M TBS (0.08 M Tris-HCl, 0.02 M Tris-base, 0.9% saline, pH 7.4) at 4 °C for cryoprotection. Brains were sectioned into 40 μm coronal sections through the entirety of the dentate gyrus using a Leica 1000S vibratome (Leica Biosystems, Deer Park, IL, USA). During sectioning, the tissue was bathed in 0.1 M TBS and kept on ice. Tissue slices were stored in antifreeze solution (0.05 M TBS, 30% ethylene glycol, and 20% glycerol) at −20 °C until immunohistochemical processing.

### 2.3. Immunohistochemistry

Peroxidase immunohistochemistry was performed on free-floating tissue in a series of every 10th section (i.e., 400 μm intervals) through the rostro-caudal extent of the dentate gyrus to visualize BrdU-labeled cells (10–12 sections per rat). Sections were rinsed in 0.1 M TBS (pH 7.4) three times for 10 min between steps unless otherwise noted. Tissue was incubated for 30 min in 0.6% H_2_O_2_ to eliminate endogenous peroxidase activity, and then DNA was denatured with 2 N HCl at 37 °C for 30 min. Next, tissue was incubated for 10 min in 0.1 M borate buffer (pH 8.5) to neutralize the acid. Tissue was next blocked for 30 min in a solution of 0.1 M TBS, 0.1% Triton-X 100, and 3.0% normal horse serum, followed by incubation for 16 h on a platform shaker at 4 °C in mouse monoclonal antibodies against BrdU (1:400 in blocking solution; Roche Diagnostics, Indianapolis, IN, USA). Sections were next incubated in horse anti-mouse secondary antibodies (1:100 in 0.1 M TBS; Vector Laboratories) for 4 h, followed by a 1.5 h incubation in avidin-biotin horseradish peroxidase solution (1:50; ABC Elite Kit, Vector Laboratories). Sections were reacted for 3–5 min in a solution of 3,3′diaminobenzidine (0.5 mg/mL; Sigma-Aldrich, Atlanta, GA, USA) and 0.003% H_2_O_2_ in 0.1 M TBS and then mounted onto Superfrost Plus microscope slides (Fisher Scientific, Suwanee, GA, USA). Dried sections were counterstained with cresyl violet acetate (0.5%), dehydrated with ethanol, cleared with xylene, and coverslipped using Permount (Sigma-Aldrich, St. Louis, MO, USA). Cresyl staining was used to visualize the GCL.

Fluorescent immunohistochemistry was used to double-label tissue sections for BrdU and neuronal nuclei (NeuN) for every 10th section through the rostro-caudal extent of the dentate gyrus. NeuN is expressed in mature neurons, and thus the co-labeling of BrdU and NeuN indicated that the newly generated cells were neurons. The protocol was identical to that used for peroxidase immunochemistry (described above) with some exceptions. Normal goat serum was used in place of NHS, and the primary antibodies consisted of mouse monoclonal antibody against NeuN (Chemicon/Millipore, Burlington, MA, USA), and rat monoclonal antibody against BrdU (Abcam, Cambridge, UK), with both at a concentration of 1:200 in blocking solution. The secondary antibody combination consisted of Alexa Fluor488 goat anti-mouse IgG and Alexa Fluor568 goat anti-rat IgG (both 1:200; Thermo Fisher Scientific, Waltham, MA, USA). Sections were mounted on Superfrost Plus slides, coverslipped with the anti-fading agent diazobicyclooctane (0.1 M TBS, 2.5% DABCO, 10% polyvinyl alcohol, and 20% glycerol), and stored at −20 °C.

### 2.4. Microscopy

**Figure 2:**
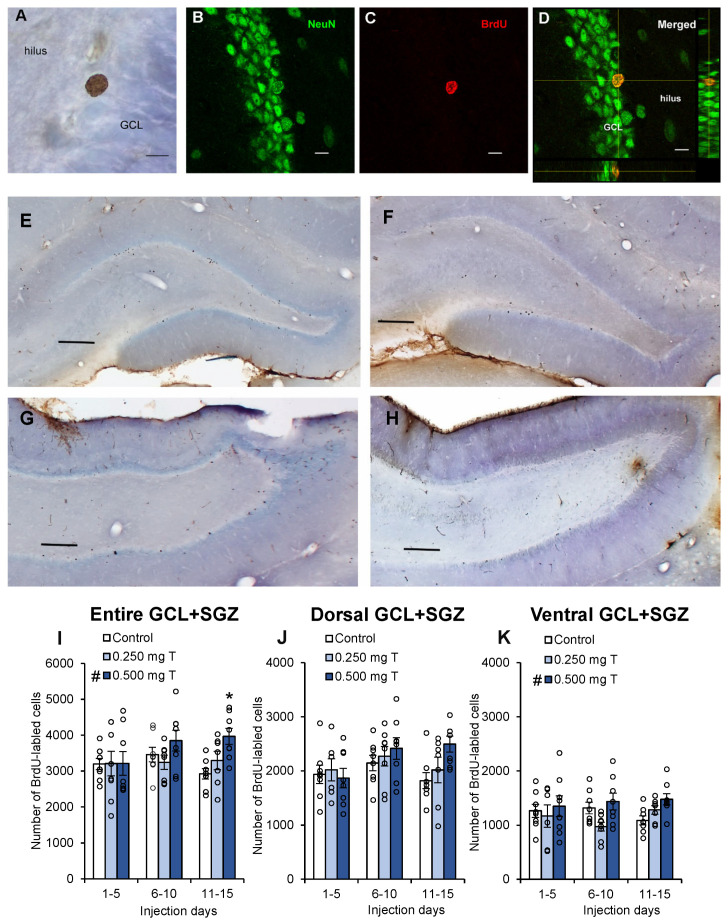
Quantification of the number of BrdU-labeled cells in the granule cell layer and subgranular zone of the dentate gyrus of adult male rats. Photomicrographs are shown for (**A**) a representative BrdU-labeled cell collected using bright-field microscopy (Zeiss Axio Imager D1; 100× Achrocplan oil-immersion lens, 1.25 NA) and (**B**–**D**) a cell double-labeled with BrdU and NeuN collected using confocal microscopy (Olympus Fluoview 3000; 60× UPlanApo oil-immersion lens, 1.5 NA). Representative photomicrographs from rats in the 10–15 day injection groups are also shown for (**E**) dorsal and (**G**) ventral sections taken from a subject injected with 0.500 mg T and for (**F**) dorsal and (**H**) ventral sections taken from a subject injected with oil. The scale bar represents 10 μm for (**A**–**D**) and 200 μm for (**E**–**H**). Graphs depict mean ± SEM number of BrdU-labeled cells estimated based on cell counts, with data for individual rats shown as circles. (**I**) For the entire dentate gyrus, there was a significant effect of testosterone dose (# *p* = 0.029), with more labeled cells in the GCL+SGZ of the 0.500 mg T group than in either of the other two groups (both *p* < 0.04) and no difference between the 0.250 mg T group and the control group. There was no significant effect of hormone injection day and no dose×day interaction. Analyses within the 5-day blocks indicated that the effect of testosterone was only significant on days 11–15 (* *p* = 0.006). (**J**) For the dorsal dentate gyrus, there was no significant effect of testosterone dose and no interaction effect (*p* = 0.36), but there was a nearly significant effect of hormone injection day (*p* = 0.084). (**K**) For the ventral dentate gyrus, there was a significant effect of dose (# *p* = 0.037), with more labeled cells in the ventral GCL+SGZ of the 0.500 mg T group than that of the 0.250 mg T group (*p* = 0.011). There was also a marginal difference between the 0.500 mg T group and the control group (*p* = 0.067). There was no significant effect of hormone injection day and no dose × day interaction.

All BrdU-labeled cells were counted in the granule cell layer (GCL) and hilus on every 10th section through the entire dentate gyrus. During the entire counting process, the experimenter was blind to the treatment groups. All cells were counted using a 100× oil immersion lens (Acroplan, 1.25 NA, Zeiss, Oberkochen, Germany) on a light microscope (Zeiss Axio Imager D1, Oberkochen, Germany). Cells observed in the SGZ, within 20 μm of the GCL, were combined with granule cell layer counts (GCL+SGZ). Cells were considered labeled if they exhibited a dark brown punctate stain (Figure 2A). To estimate the total number of BrdU-labeled cells in the brain, the final cell counts were multiplied by ten (inverse of the sampling ratio). Labeled cells were counted in the hilus and compared to counts in the GCL+SGZ to determine whether any experimentally induced effects influenced cell division in the brain more broadly rather than being specific to the neurogenic niche along the SGZ. Progeny from progenitor cells in the hilus give rise to a population of ectopic cells that are morphologically and physiologically distinct from the granule cells produced along the SGZ [91,92]. Sections were also categorized as dorsal (interaural 3.70–7.20 mm) or ventral (interaural 3.70–2.20 mm) [93], because there is evidence that these regions serve different functions [94].

Confocal microscopy (Olympus Fluoview 3000, Tokyo, Japan) was used to determine if BrdU-labeled cells co-localized with the NeuN label (Figure 2B–D). For each brain, 50 BrdU-labeled cells were selected from sections throughout the hippocampus, with no more than 10 cells sampled from the same section, and sections were evenly sampled throughout the rostral-caudal extent of each brain. This sampling method has been previously used by our labs [78,87]. Z-stack images were collected for each cell using a 60 × lens (UPlanApo, 1.5NA, Olympus, Tokyo, Japan) at interval step sizes of 0.7 μm. Image intensity was assessed using ImageJ software (ver. 1.53t; National Institutes of Health, Bethesda, MD, USA). BrdU-labeled cell boundaries were marked for the red label, and the intensity was determined for the green label (NeuN) at the same location. Using an area the same size as each BrdU-labeled cell, background intensity was determined in a nearby region lacking cell bodies within the hilus. A cell was considered double-labeled if its intensity was greater than or equal to three times the background intensity. The percentage of BrdU-labeled cells co-localized with NeuN was calculated for each brain.

Photomicrographs were collected using a 2.5× lens (Zeiss A-Plan, 0.06 NA, Oberkochen, Germany) on a light microscope (Zeiss Axio Imager D1; AxioCam 208 color, Oberkochen, Germany) with imaging software (ZEN 3.6, Zeiss, Oberkochen, Germany) for all the cresyl-stained brain sections. ImageJ (ver. 1.53t; National Institutes of Health, Bethesda, MD, USA) was used to measure the areas of the GCL+SGZ and hilus for each digital image. Cavalieri’s principle [95] was used to estimate volumes (mm^3^) of the GCL+SGZ and hilus: areas area were summed (mm^2^) and multiplied by the distance between sections (0.4 mm).

### 2.5. Statistical Analysis

For all statistical analyses, “dose” and “day” (i.e., hormone injection period) were fixed factors. For each experiment, the total number of BrdU-labeled cells in the GCL+SGZ and the hilus were analyzed separately using univariate ANOVAs. Given past evidence that sex steroids enhanced neurogenesis during specific time periods in male rodents [89], we ran planned (*a priori*) univariate ANOVAs to assess the effects of steroids within each 5-day block of neuronal development. The data were then divided into dorsal and ventral regions of the dentate gyrus [93], and each region was analyzed separately using univariate ANOVAs. The percentage of BrdU and NeuN double-labeled cells in the GCL+SGZ were analyzed using a univariate ANOVA for each experiment. The estimated volumes of the GCL+SGZ and hilus were also analyzed using univariate ANOVA. The Shapiro–Wilk tests were used to confirm normal distributions for all variables and groups (all *p* > 0.10), and skewness and kurtosis were also assessed (Table S1). All statistical tests were carried out using IBM SPSS (version 28.0) with α = 0.05.

## 3. Results

For the entire dentate gyrus, there was a significant effect of testosterone dose (Figure 2I; *F*_2,60_ = 3.75, *p* = 0.029), with more BrdU-labeled cells in the GCL+SGZ of the 0.500 mg T group than that in either of the other two groups (both *p* < 0.04) and no significant difference between the 0.250 mg T group and the control group (*p* = 0.77). There was no significant effect of hormone injection day (*p* = 0.29) and no dose×day interaction (*p* = 0.22). However, analyses within each block revealed no significant effect of testosterone on the number of BrdU-labeled cells during days 1–5 (*p* = 0.99) or days 6–10 (*p* = 0.19). There was a significant effect of testosterone dose on days 11–15 (*F*_2,20_ = 6.68, *p* = 0.006), with the 0.500 mg T group having significantly higher cell counts than both of the other two groups (*p* < 0.035), but no difference between the 0.25 mg T group and the oil-injected control group (*p* = 0.23). Thus, the strongest effects of testosterone were in the later stage of neuronal development.

For the dorsal dentate gyrus (Figure 2J), there was no significant effect of testosterone dose (*p* = 0.14) and no interaction effect (*p* = 0.29), but there was a nearly significant effect of hormone injection day (*p* = 0.084) with days 6–10 showing more labeled cells than during the other two five-day blocks. For the ventral dentate gyrus (Figure 2K), there was a significant effect of testosterone dose (*F*_2,60_ = 3.50, *p* = 0.037), with more labeled cells in the ventral GCL+SGZ of the 0.500 mg T group than that of the 0.250 mg T group (*p* = 0.011). There was also a marginal difference between the 0.500 mg T group and the control group (*p* = 0.067). There was neither a significant effect of hormone injection day (*p* = 0.93) nor a dose×day interaction (*p* = 0.32) for the ventral GCL+SGZ. For the testosterone experiment, there were no significant effects upon number of BrdU-labeled cells observed in the hilus regardless of which region of the dentate gyrus was analyzed (all *p* > 0.18).

**Figure 3:**
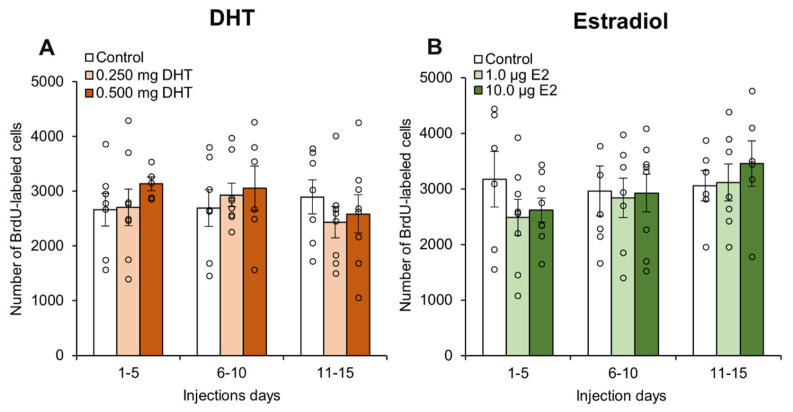
Mean (±SEM) number of BrdU-labeled cells in the granule cell layer and subgranular zone of the dentate gyrus of adult male rats, with data for individual rats shown as circles. (**A**) There were no significant effects of daily DHT injections, and (**B**) there was also no significant effect of daily estradiol injections. In both experiments, there was no significant effect of the day of injection and no significant interaction effect.

Broadly, DHT injections had no effect on the number of BrdU-labeled cells observed (Figure 3A). For the entire dentate gyrus, there were no significant effects of hormone injection day (*p* = 0.567), drug dose (*p* = 0.653), or an interaction effect (*p* = 0.687) for total number of BrdU-labeled cells in the GCL+SGZ. There were also no significant effects upon total number of BrdU-labeled cells in the hilus for the entire dentate gyrus (all *p* > 0.41). There were also no significant effects for the total number of BrdU-labeled cells in the dorsal GCL+SGZ (all *p* > 0.56) or the ventral GCL+SGZ (all *p* > 0.57). Additionally, there were no significant effects for the total number of BrdU-labeled cells in the dorsal hilus (all *p* > 0.28) or the ventral hilus (all *p* > 0.16).

Estradiol injections also had no effect on the number of BrdU-labeled cells (Figure 3B). For the entire dentate gyrus, there were no significant effects of hormone injection day, (*p* = 0.317), drug dose (*p* = 0.671), or an interaction effect (*p* = 0.757) for total number of BrdU-labeled cells in the GCL+SGZ. Similarly, there were no significant effects upon total number of BrdU-labeled cells in the hilus when considering the entire dentate gyrus (all *p* > 0.75). There were also no significant effects for the total number of BrdU-labeled cells in the dorsal GCL+SGZ (all *p* > 0.45) or the ventral GCL+SGZ (all *p* > 0.28). Finally, there were no significant effects for the total number of BrdU-labeled cells in the dorsal hilus (all *p* > 0.74) or the ventral hilus (all *p* > 0.57).

In general, 70–90% of BrdU-labeled cells in each subject expressed the neuronal marker NeuN, indicating that BrdU-labeling was a useful index of neurogenesis levels (Table 1). There were no significant effects of steroid hormone treatments, hormone injection day, or interaction effects for any of the three experiments (testosterone: all *p* > 0.13; DHT all *p* > 0.81; estradiol all *p* > 0.32). The volume of the GCL+SGZ was relatively consistent across groups within each experiment (Table 2), with no significant effects of steroid hormone treatments, hormone injection day, or interaction effects for any of the three experiments (testosterone: all *p* > 047.; DHT: all *p* > 0.23; estradiol: all *p* > 0.20). Finally, the volume of the hilus showed no significant effects of steroid hormone treatments, hormone injection day, or interaction effects for any of the three experiments (Table 2; testosterone: all *p* > 0.07; DHT: all *p* > 0.10; estradiol: all *p* > 0.54).

## 4. Discussion

Here we found that a high dose of testosterone (0.500 mg/rat) increased neurogenesis in the adult male hippocampus. This result corroborates past evidence that testosterone enhances the survival of new hippocampal neurons [74,78,82,85]. When the testosterone data were divided by regions of the hippocampus, only the ventral region showed a significant effect, which suggests that the effects of testosterone upon neuron survival may be specific to the ventral hippocampus. Planned comparisons within each developmental stage revealed that the effects of testosterone were significant only during days 11–15 (Figure 2I). Surprisingly, we found no effects of DHT or estradiol injections upon hippocampal neurogenesis regardless of the stage in the neuron maturation cycle when hormones were injected. Most past studies have also found no effect of estradiol upon neurogenesis in male rodents [74,88,96]. There are, however, a number of studies that have demonstrated that DHT enhances neurogenesis [74,85,86,87]. Our contradictory results may be explained by the timing of injections and/or the dose of DHT that was used. A majority of BrdU-labeled cells were found to be neurons, and none of our treatments significantly changed the likelihood that BrdU-labeled cells would differentiate into neurons (Table 1).

### 4.1. Testosterone May Enhance Neurogenesis During a Critical Period

Our results add to growing evidence that testosterone enhances neurogenesis through a change in cell survival rather than cell proliferation. Past studies have shown that neither castration nor testosterone supplementation influence the number of newly proliferated cells in the dentate gyrus 24 h after BrdU injection [74,80,83,97] or based on the endogenous cell proliferation marker Ki67 [75,76,79,82,84]. We also observed a minimal effect of testosterone injections during days 1–5 after BrdU injection, when effects upon cell proliferation could have occurred [59]. We did observe an overall enhancement in the number of BrdU-labeled cells irrespective of time period by the higher dose of testosterone, which is supported by some past results. Among both rats and mice, 30–35 days of testosterone supplementation increased neurogenesis [74,82,85], but shorter periods of testosterone supplementation (15–21 days) had no effect [78,79,80]. Although castration caused a decrease in neurogenesis 15 days after castration, 15 days of injections with the same dose of testosterone that we used (0.500 mg/day) surprisingly had no effect on neurogenesis among castrated male rats [78]. This suggests that a more acute 5-day period of testosterone delivery, especially later in development, may have a different effect on neurogenesis compared to 15 consecutive days of hormone injections. An alternative interpretation is that a longer (10-day) period without exposure to testosterone may have sensitized the newly developing neurons to the effects of testosterone in some way. Furthermore, current evidence suggests that even later stages of development (15–30 days after mitosis) may be more strongly influenced by testosterone, and this should be assessed in future work. The period during which testosterone seems to have the strongest effects upon cell survival (11–30 days after birth) corresponds with the period during which GABAergic input from neighboring cells within the dentate gyrus transitions from being excitatory to inhibitory [98,99], and GABAergic signaling promotes neuronal maturation [100,101]. This leads to the possibility that testosterone or its metabolites are influencing neurogenesis by modifying GABA signaling during a critical period of development [102].

Our results suggest that testosterone has a stronger effect in the ventral hippocampus than in the dorsal hippocampus. The dorsal and ventral hippocampus have different neural connections and gene expression levels [94,103], and the dorsal hippocampus mainly regulates spatial learning and memory, whereas the ventral hippocampus mainly regulates stress and anxiety [104,105,106]. There is some evidence that social stress selectively decreases neurogenesis in the ventral hippocampus [107,108,109]. For example, social instability within male rat dominance hierarchies caused a decrease in neurogenesis, but only in the ventral dentate gyrus [109]. Selectively ablating neurogenesis in the ventral or dorsal dentate gyrus revealed that neurogenesis within the dorsal region was necessary for contextual discrimination (a spatial task), whereas neurogenesis in the ventral region was necessary for the anxiolytic effects of antidepressants [110]. Interestingly, there is evidence that testosterone can suppress glucocorticoids [111,112] and reduce anxiety among male rats [113,114]. Given that glucocorticoids suppress adult neurogenesis [115,116], testosterone’s effects upon neurogenesis and anxiety may involve either direct actions upon the hippocampus or indirect effects upon the hypothalamic–pituitary–adrenal axis.

### 4.2. Estradiol and DHT Had No Effect on Neurogenesis

We observed no significant effect of estradiol upon hippocampal neurogenesis, which supports most past findings [74,88,96,117], but one study following an experimental protocol similar to ours found that estradiol injections given 6–10 days after BrdU injection caused an increase in neurogenesis [89]. This discrepancy may be due to a species difference, as the previous study involved meadow voles. Rats have higher levels of adult neurogenesis than mice and the neurons mature more rapidly [118], suggesting that neurogenesis in voles might also follow a different developmental timeline. In support of this idea, only about 65% of cells were found to be neurons in the vole experiment [89] compared to about 90% in our experiment. This suggests that neurons may mature more slowly in voles than rats. This would suggest that the effects of estradiol on development in rats should have occurred earlier in development, but we found no effects of estradiol on any stage of development. It remains possible that a shorter period of estradiol exposure early in neuron development could enhance neurogenesis in male rats. Another variable to consider is dosing. Our higher dose (10 μg/rat) resulted in serum estradiol that was about ten-fold higher than among intact male rats [74], which is why we also used a lower dose (1 μg/rat). The experiment with voles also used a 10 μg dose, but voles have about 1/10th the body mass of rats, suggesting that the dosing was likely supraphysiological for the voles. Perhaps supraphysiological doses of estradiol would also enhance neurogenesis in male rats, but this would not provide a reasonable explanation for how physiological doses of testosterone enhance neurogenesis. Interestingly, castration has no significant effect on hippocampal E2 levels in male rats [119]. This suggests that that the reduction in neurogenesis observed in castrated rats [74,77] is also unlikely to occur through an estrogen-dependent pathway.

Most current evidence suggests that testosterone influences adult neurogenesis through an androgenic pathway [120], so it was surprising that we did not observe any effect of DHT upon neurogenesis. Specifically, a number of past studies with rats and mice have shown that prolonged DHT exposure increases neurogenesis in castrated males [74,85,86,87], and the experiments with rats used the same DHT doses that were used in the present study. In contrast to our experiment, all past studies have involved prolonged (30–37 days) exposure to DHT, so this suggests that short-term exposure to DHT does not enhance neurogenesis. However, this does not explain why we observed enhanced neurogenesis with 5 days of testosterone injections but not 5 days of DHT injections. One explanation for our results is that we may have been using supraphysiological doses of DHT. We used the same doses of testosterone and DHT (0.25 and 0.50 mg/rat), but it seems unlikely that all testosterone that reaches the brain would normally be converted to DHT [121,122]. The over-expression of androgen receptors in neural tissue of transgenic mice eliminated the neurogenesis-enhancing effects of DHT [86], suggesting that supraphysiological dosing with androgens would not enhance hippocampal neurogenesis. However, dosing may not provide a full explanation for our results, as the same doses of DHT that we used were used in past experiments with castrated rats that resulted in increased neurogenesis after prolonged exposure [74,85,87]. Another explanation is that a combination of estrogenic and androgenic effects is required for an acute dose of testosterone to enhance neurogenesis. Newly proliferated cells in the dentate gyrus express estrogen receptors but not androgen receptors [70,85], suggesting that estrogens could act directly on newly proliferated cells, whereas the effects of androgens may be indirect, involving other layers of the hippocampus or even other brain regions.

## 5. Conclusions

Our results refine our understanding of how testosterone influences the maturation of new adult neurons in the hippocampus. In summary, we found that a high physiological dose of testosterone given over a short period (5 days) enhanced adult neurogenesis selectively within the ventral hippocampus. It remains unclear whether this effect involves an estrogenic pathway, an androgenic pathway, or perhaps synergistic effects of both pathways. Future work could use the dose of testosterone that is known to enhance neurogenesis (0.5 mg/rat) in combination with androgen and/or estrogen receptor antagonists to further assess the molecular mechanisms behind testosterone’s effects on neurogenesis. Our results also suggest that testosterone may have a stronger effect on the later stages of neurogenesis, and there is some evidence that cells that are about 11–15 days old may be critical for memory formation [61,62,63,64,65,66]. Future work could investigate even later stages of neurodevelopment, particularly in the 15–30 day age range for rodents, as these older cells seem to be important for some forms of memory [66,123].

## Figures and Tables

**Table 1:** Percentage (mean ± SEM) of BrdU-labeled cells in the GCL+SGZ that were co-labeled with NeuN, with 50 cells analyzed on a confocal microscope for each subject.

Hormone	Days	Dose	*n*	Percent Cells Co-Labeled
Testosterone		Control	8	83.00 ± 5.38
	1–5	0.250 mg/rat	6	78.67 ± 9.42
		0.500 mg/rat	8	76.88 ± 5.24
		Control	8	75.38 ± 5.92
	6–10	0.250 mg/rat	8	87.75 ± 4.18
		0.500 mg/rat	8	74.13 ± 9.76
		Control	8	87.11 ± 2.35
	11–15	0.250 mg/rat	7	89.43 ± 2.78
		0.500 mg/rat	8	86.75 ± 4.09
DHT		Control	7	80.14 ± 4.40
	1–5	0.250 mg/rat	8	81.38 ± 3.70
		0.500 mg/rat	5	82.80 ± 8.00
		Control	7	80.57 ± 7.50
	6–10	0.250 mg/rat	8	85.63 ± 3.89
		0.500 mg/rat	6	82.17 ± 7.02
		Control	7	87.14 ± 4.41
	11–15	0.250 mg/rat	8	80.25 ± 4.31
		0.500 mg/rat	8	82.88 ± 4.45
Estradiol		Control	6	92.00 ± 1.26
	1–5	1.0 μg/rat	6	91.25 ± 1.31
		10.0 μg/rat	8	91.25 ± 3.16
		Control	7	92.29 ± 1.48
	6–10	1.0 μg/rat	8	82.86 ± 6.98
		10.0 μg/rat	8	92.25 ± 1.78
		Control	6	91.33 ± 3.00
	11–15	1.0 μg/rat	8	93.71 ± 2.16
		10.0 μg/rat	6	93.33 ± 1.76

**Table 2:** Volumes (mean ± SEM) of the combined granule cell layer and subgranular zone (GCL+SGZ) and hilus for each group of rats from all three experiments.

Hormone	Days	Dose	GCL+SGZ (mm^3^)	Hilus (mm^3^)
Testosterone		Control	2.074 ± 0.252	5.141 ± 0263
	1–5	0.250 mg/rat	1.725 ± 0.182	4.806 ± 0.545
		0.500 mg/rat	2.150 ± 0.085	4.984 ± 0.116
		Control	1.847 ± 0.138	5.305 ± 0.321
	6–10	0.250 mg/rat	1.857 ± 0.120	4.578 ± 0.286
		0.500 mg/rat	2.044 ± 0.197	4.989 ± 0.440
		Control	1.956 ± 0.135	5.168 ± 0.194
	11–15	0.250 mg/rat	1.960 ± 0.090	5.576 ± 0.334
		0.500 mg/rat	1.996 ± 0.116	5.734 ± 0.268
DHT		Control	1.835 ± 0.106	3.997 ± 0.209
	1–5	0.250 mg/rat	1.829 ± 0.068	4.303 ± 0.320
		0.500 mg/rat	1.996 ± 0.128	3.756 ± 0.122
		Control	1.841 ± 0.103	3.633 ± 0.202
	6–10	0.250 mg/rat	1.935 ± 0.114	4.397 ± 0.247
		0.500 mg/rat	2.117 ± 0.063	4.538 ± 0.388
		Control	1.834 ± 0.124	4.049 ± 0.190
	11–15	0.250 mg/rat	1.938 ± 0.101	4.278 ± 0.260
		0.500 mg/rat	1.866 ± 0.130	4.229 ± 0.164
Estradiol		Control	1.670 ± 0.060	3.724 ± 0.169
	1–5	1.0 μg/rat	1.641 ± 0.055	3.771 ± 0.212
		10.0 μg/rat	1.751 ± 0.063	3.919 ± 0.220
		Control	1.612 ± 0.078	3.881 ± 0.169
	6–10	1.0 μg/rat	1.596 ± 0.104	3.804 ± 0.327
		10.0 μg/rat	1.650 ± 0.118	3.681 ± 0.146
		Control	1.558 ± 0.087	3.355 ± 0.164
	11–15	1.0 μg/rat	1.540 ± 0.223	3.601 ± 0.348
		10.0 μg/rat	1.730 ± 0.099	3.884 ± 0.180

## Data Availability

The data presented in this study are openly available in Data @Middlebury (https://www.middlebury.edu/midddata, accessed 15 January 2025).

## Supplementary Materials

The following supporting information can be downloaded at: https://www.mdpi.com/article/10.3390/biom15040542/s1, Table S1: Skewness and Kurtosis for the total number of BrdU-labeled cells in the granule cell layer and subgranular zone for each group of rats from all three experiments.
