# Supplementary material for: Effects of Testosterone and Its Major Metabolites upon Different Stages of Neuron Survival in the Dentate Gyrus of Male Rats
Source: Biomolecules. 2025 Apr 7;15(4):542. doi: 10.3390/biom15040542 (PMC12024780; doi:10.3390/biom15040542)
Supplement: Supplementary file 1 [file biomolecules-15-00542-s001.zip › biomolecules-3455542-supplementary.pdf]

Supplementary Materials

Table S1. Skewness and Kurtosis for the total number of BrdU-labeled cells in the granule cell layer and subgranular zone for each group of rats from all three experiments.

Hormone	Days	Dose	Skewness	Kurtosis
Testosterone		Control	0.150	−0.610
	1–5	0.250 mg/rat	0.364	−1.193
		0.500 mg/rat	0.865	−1.341
		Control	0.127	0.166
	6–10	0.250 mg/rat	−0.266	−1.743
		0.500 mg/rat	0.190	−0.453
		Control	−0.027	−0.197
	11–15	0.250 mg/rat	−0.657	−1.000
		0.500 mg/rat	0.008	−1.559
DHT		Control	−0.152	−0.343
	1–5	0.250 mg/rat	0.410	−0.129
		0.500 mg/rat	0.429	−1.116
		Control	−0.259	−1.203
	6–10	0.250 mg/rat	1.037	−0.197
		0.500 mg/rat	−0.389	−0.823
		Control	−0.348	−1.768
	11–15	0.250 mg/rat	0.935	1.342
		0.500 mg/rat	0.107	0.363
Estradiol		Control	−0.422	−1.893
	1–5	1.0 µg/rat	−0.142	−0.275
		10.0 µg/rat	−0.135	1.418
		Control	1.021	0.794
	6–10	1.0 µg/rat	−0.526	−0.952
		10.0 µg/rat	−0.561	−1.436
		Control	−0.743	0.740
	11–15	1.0 µg/rat	0.209	−1.385
		10.0 µg/rat	−0.683	1.412
